# Autophagy Is Polarized toward Cell Front during Migration and Spatially Perturbed by Oncogenic Ras

**DOI:** 10.3390/cells10102637

**Published:** 2021-10-02

**Authors:** Manish Kumar Singh, Giulia Zago, Irina Veith, Jacques Camonis, Mathieu Coppey, Maria Carla Parrini

**Affiliations:** 1 Institut Curie, Centre de Recherche, Paris Sciences et Lettres Research University, 75005 Paris, France; manish.singh@pasteur.fr (M.K.S.); ZagoG@mskcc.org (G.Z.); Irina.Veith@curie.fr (I.V.); jacquescamonis@gmail.com (J.C.); Mathieu.Coppey@curie.fr (M.C.); 2 Institut National de la Santé et de la Recherche Médicale (INSERM), U830, 75005 Paris, France; 3 Centre National de la Recherche Scientifique (CNRS), UMR168, Sorbonne University, 75005 Paris, France

**Keywords:** autophagy, migration, cancer, Ras, micro-patterns

## Abstract

Autophagy is a physiological degradation process that removes unnecessary or dysfunctional components of cells. It is important for normal cellular homeostasis and as a response to a variety of stresses, such as nutrient deprivation. Defects in autophagy have been linked to numerous human diseases, including cancers. Cancer cells require autophagy to migrate and to invade. Here, we study the intracellular topology of this interplay between autophagy and cell migration by an interdisciplinary live imaging approach which combines micro-patterning techniques and an autophagy reporter (RFP-GFP-LC3) to monitor over time, during directed migration, the back–front spatial distribution of LC3-positive compartments (autophagosomes and autolysosomes). Moreover, by exploiting a genetically controlled cell model, we assessed the impact of transformation by the Ras oncogene, one of the most frequently mutated genes in human cancers, which is known to increase both cell motility and basal autophagy. Static cells displayed an isotropic distribution of autophagy LC3-positive compartments. Directed migration globally increased autophagy and polarized both autophagosomes and autolysosomes at the front of the nucleus of migrating cells. In Ras-transformed cells, the front polarization of LC3 compartments was much less organized, spatially and temporally, as compared to normal cells. This might be a consequence of altered lysosome positioning. In conclusion, this work reveals that autophagy organelles are polarized toward the cell front during migration and that their spatial-temporal dynamics are altered in motile cancer cells that express an oncogenic Ras protein.

## 1. Introduction

Autophagy is an evolutionarily conserved physiological process through which dispensable or dangerous cellular components, such as damaged proteins or organelles, are captured into double-membrane organelles called autophagosomes and then brought to lysosomes to be degraded and recycled for other uses [1]. Autophagosomes can originate from several different membrane compartments and traffic through the cell to fuse with lysosomes, to form autolysosomes, or with other endomembrane compartments [2]. During autophagy, the cytosolic protein LC3 (microtubule-associated protein 1A/1B-light chain 3) is recruited to autophagosomal membranes, making it a widely used marker of autophagosomes and autolysosomes [3].

Defects in autophagy have been linked to numerous human diseases, especially neurodegenerative, inflammatory disorders and cancer [4]. In cancer, autophagy has a complex and context-dependent role: it hinders early cancer development, but it facilitates progression of advanced tumors [5,6]. In Ras-mutated cancers, the basal autophagy levels are enhanced and support Ras-driven transformation [7,8,9].

Several investigations reported a role of autophagy in promoting cell motility, invasion, and consequent metastasis [5,6]. Many cancer cells require autophagy to invade; for example, inhibition of autophagy was shown to impair invasion of triple-negative breast cancer cells [10]. However, the exact molecular mechanisms linking autophagy and motility/invasion are still poorly understood. A first possible mechanism involves the autophagy-triggered secretion of pro-migratory molecules, including the cytokine IL-6 and the metalloproteinase MMP2 [11,12]. A second possible mechanism involves the stimulation, by selective autophagy, of the dynamic turnover of integrin-based focal adhesion sites during motility, via the autophagy cargo adapter NBR1 [13] and/or via a direct interaction between LC3B at the autophagosome and the focal adhesion protein paxillin [14]. Again, the role of autophagy appears to be context-dependent, because a few works reported that autophagy might as well inhibit motility/invasion in specific cell models (including MEFs) [15,16,17].

Seeking the principles of the spatial-temporal organization linking autophagy and cell motility, we precisely quantified the topology and the spatial dynamics of autophagy compartments during motility. In order to impose a chosen directionality, we seeded cells on fibronectin line micro-patterns [18,19,20,21]. The location and density of autophagosomes, as well of autolysosomes, were tracked with the well-established reporter (RFP-GFP-LC3B) [3,22]. Finally, to evaluate the impact of oncogenic Ras on autophagy topology in a genetically controlled cell model, we compared isogenic normal HEK-HT cells (human embryonic kidney cells, immortalized but not transformed) and transformed HEK-HT-H-RasV12 cells (tumorigenic, invasive, and metastatic, due to expression of constitutive active H-RasV12) [23,24]. Consistently with previous reports on Ras-mutated cancer cells [7,8,9], the transformation of HEK-HT cells by oncogenic H-RasV12 leads to increased autophagy [25].

## 2. Materials and Methods

### 2.1. List of Plasmids

pmRFP-EGFP-rLC3 was used to express the autophagy reporter [3,22].

### 2.2. List of Reagents

Dulbecco’s modified Eagle’s medium (SH30081.01) was purchased from Cytiva- Fischer scientific (Waltham, MA, USA). Earle’s Balanced Salt Solution (24010043), phosphate-buffered saline (10010015), l-glutamine (25030024), penicillin-streptomycin (15140122), sodium pyruvate (11360070), and Geneticin (10131035) were purchased from Gibco Life Technologies (Dun Laoghaire, Dublin, Ireland). Fetal bovine serum (FB-1003/500) was purchased from Biosera (Nuaille France). Hygromycin B Gold (ant-hg), Zeocin (ant-zn), and puromycin (ant-pr) were purchased from InvivoGen (San Diego, CA, USA). Bovine serum albumin (04-100-812-C) was purchased from Euromedex (Souffelweyersheim France). jetPRIME transfection buffer (712-60) and jetPRIME transfection reagent (114-07) were purchased from Polyplus (Illkirch-Graffenstaden France). Lysotracker (PHE0023) was purchased from Invitrogen (Waltham, MA, USA). Fibronectin (PHE0023) was purchased from Life Technologies (Carlsbad, CA, USA).

### 2.3. Cell Culture and Transfections

HEK-HT and HEK-HT-H-RasV12 cells [23,24] were obtained from Christopher Counter laboratory (Duke University, Durham, NC, USA). Cells were cultured in DMEM medium containing 10% FBS, 1% sodium pyruvate, 1% penicillin-streptomycin, and 1% l-glutamine at 37 °C and 5% CO_2_. For HEK-HT cells, hygromycin (100 μg/mL) and Geneticin (400 μg/mL) were added in culture media. For HEK-HT-H-RasV12, hygromycin, Geneticin, and Zeocin (300 µg/µL) were added in cell culture media.

Cells were transfected with plasmid DNA using jetPRIME transfection reagent according to the manufacturer’s protocol. Cells were seeded in 6-well plates at a density of 0.5 × 10^6^ cells per well; 24 h later, transfection mix containing 2 μg plasmid DNA, 200 μL of jetPRIME buffer, and 4 μL of jetPRIME reagent were added dropwise to the cells; medium was changed 4 h later. Cells were used for experiments 24 h post DNA transfection.

### 2.4. Surface Patterning

Eighteen millimeter glass coverslips were used to print micro-patterns following a previously published protocol [18]. A defined 10 μm thick line and 45 μm long crossbow shapes were micro-patterned, using PLL-g-PEG (Surface Solutions Switzerland), an UV lamp (UV ozone oven, 185 nm equipped with ozone catalyzer, UVO cleaner, model 342-220; Jelight), and a chrome mask (Toppan). These micro-patterned coverslips were subsequently coated with bovine fibronectin (Life Technologies, Carlsbad, CA, USA) at 10 μg/mL concentration for 1 h at 37 °C. Cells were seeded on fibronectin coated coverslips for 1 h before using for live imaging.

### 2.5. Sample Preparation

Cells were trypsinized, washed, and seeded on the patterned coverslips. Twenty thousand cells were diluted in 2 mL culture medium in 6-well plates and incubated for 1 h so that cells attached to the micro-pattern efficiently. Cells were then washed with PBS to remove unbound cells, and the culture medium was replaced by a fresh medium containing SiR-DNA (cat #: SC007, Spirochrome) at 500 nM concentration to stain the nucleus, and incubated for 0.5–1 h. The coverslips were transferred to Chamlide magnetic chambers (cat#: CM-B18-1PB) and 1 mL of medium containing SiR-DNA was added to the chamber. Cells were imaged approximately 2 h after seeding.

### 2.6. Image Acquisition

To quantify autophagy with the RFP-GFP-LC3 tandem reporter, cells were imaged using an inverted spinning disk confocal microscope from Nikon equipped with sCMOS Prime95B detector (Photometrics) with pixel size = 11 µm; 60× oil objective with NA = 1.4; laser system - 405 - 491 - 561 - 642 (50 mW - 100 mW - 50 mW - 100 mW); CSU-X1 confocal scanner unit (Yokogawa, Tokyo, Japan); MetaMorph image acquisition software (v7.10, Nashville, TN, USA); CO_2_ and temperature control unit. Images were acquired over a 2 h duration with 30 min time intervals. Images were acquired in four channels: GFP (λ_Abs_ = 491 nm, λ_Em_ = 520 nm), RFP (λ_Abs_ = 561 nm, λ_Em_ = 583 nm), SiR-DNA (CY5) (λ_Abs_ = 642 nm, λ_Em_ = 674 nm), and transmission image.

To measure the speed of cells, transmission images were acquired using a Leica DMIRE2 (Wetzlar, Germany) inverted microscope equipped with a 20× objective. Cells were tracked using an ImageJ plugin called Manual Tracking (by Fabrice Cordolières) [26].

### 2.7. Image Processing with TopoAutophagy Macro

The TopoAutophagy semi-automated tool was developed to be used with ImageJ software [26] (v1.53, Bethesda, MD, USA). The macro can be downloaded from: https://github.com/mformanu9/TopoAutophagy/blob/master/TopoAutophagy.ijm, accessed on 24 September 2021.

A stack of all the time points’ and channels’ (GFP, RFP, CY5, transmission) images was used as an input file for this macro. The first step is to orient the cell along the x-axis; the user is asked to draw a line along the direction of migration. The following step is the segmentation of LC3 compartments, cells, and nuclei. To segment the LC3 compartments (GFP, RFP), a Gaussian blur filter was used, followed by thresholding; morphological operations, such as fill holes and close, were used to refine the image. To segment the cells, the GFP, RFP, or transmission channel was used, depending on the image quality. To segment the nuclei, the CY5 channel was used; the centroid nucleus was identified at each time point to measure the distance traveled by the cell and to split the cell into 2 halves (front and back) and then further to split these 2 halves into 5 portions of equal width. For each portion, autophagosome area and count and autolysosome area and counts were measured. We only considered those cells which were migrating in only one direction throughout the duration of acquisition; the turning cells were discarded.

### 2.8. Tumor-on-Chip 3D Motility Assay

Cells were embedded in a hydrogel composed of bovine collagen type I (PureCol, Advanced Matrix, #5005) at a final concentration of 2.3 mg/mL, within PDMS-based microfluidic devices, as previously described [27]. Time-lapse phase-contrast images of tumor-on-chip were taken every 30 min for 24 h using an automated inverted DMi8 Leica microscope (Wetzlar, Germany).

For manual cell tracking analysis, we used the ImageJ macro MtrackJ [28]. The coordinates of all cell tracks were exported to an Excel file. Then, we used the open-source program DiPer [29] to generate plot to origin graphs and to compute cell speed, directionality, and directional persistence. Specifically, we ran the following Excel macros: Plot_At_Origin, Speed, DirRatio, and Autocorrel.

### 2.9. Statistical Analysis

Results are shown as mean ± standard error of the mean (SEM). Graphs were created and statistical analysis was performed using Graphpad Prism (v8.0, San Diego, CA, USA). The specific statistical tests are indicated in the legends. *p*-values less than 0.05 were considered significant.

## 3. Results and Discussion

### 3.1. Ras-Transformation Promotes an Active Random Motility in 3D Collagen Gel

**Figure 1:**
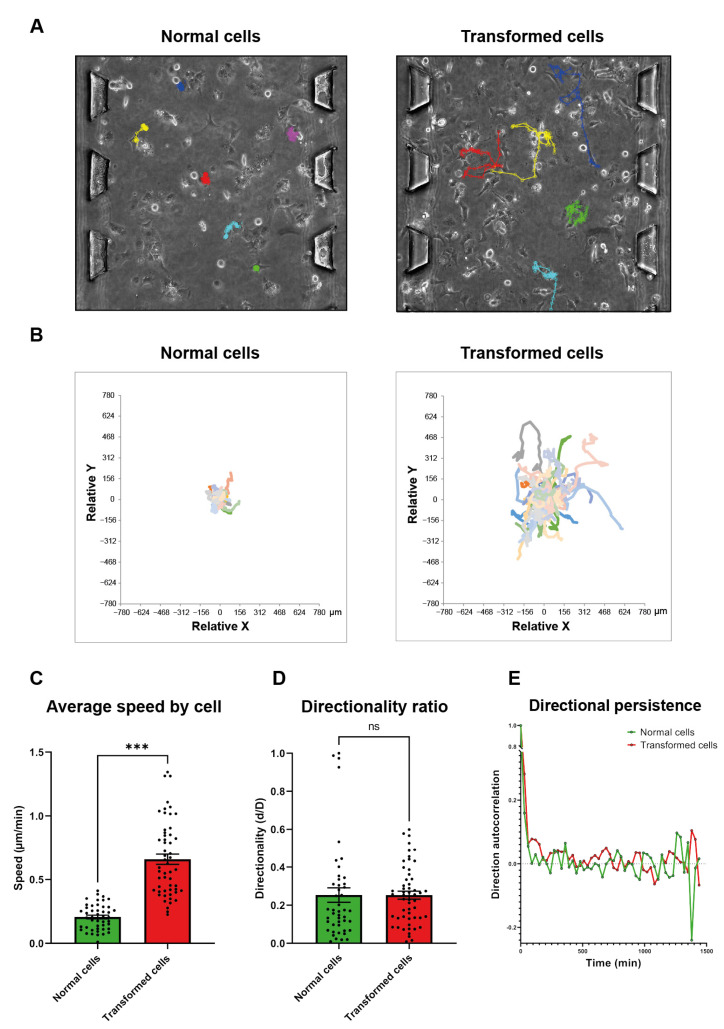
Ras-transformation promotes an active random motility in 3D collagen gel. (**A**) Representative manual tracks over 24 h of normal cells (left) and transformed cells (right) embedded in a 3D gel in a microfluidic device. The micro-pillars allow the gel’s confinement within the central channel. (**B**) Plot to origin of tracks of normal cells (left, 42 cells) and transformed cells (right, 54 cells). (**C**) Quantifications of average speed by cell of normal and transformed cells. (**D**) Quantifications of normal and transformed cells’ directionality (d/D) ratio, where d is the shortest linear distance from the start to the endpoint and D is the total track distance traveled by a cell. (**E**) Quantifications of directional persistence over time in normal cells and transformed cells, calculated as the autocorrelation function of the difference of the angles of displacement vectors between adjacent time intervals Δt (30 min). Graphs show the mean ± SEM from one experiment of *n* = 42 normal cells and *n* = 54 transformed cells. For statistics, the Mann–Whitney test was used *** *p*-value < 0.001, ns: not significant).

We studied the random 3D motility of HEK-HT cells and HEK-HT-H-RasV12 cells using a tumor-on-chip assay [27]. These isogenic cells were embedded in a collagen gel, confined in a microfluidic device, and visualized by time-lapse phase-contrast microscopy for 24 h (Figure 1A, Movies S1 and S2). Since the height of the gel was only 150 μm, cell motility was followed as pseudo-2D. Cell tracking analysis showed that Ras-transformation promotes motility (Figure 1B); HEK-HT-H-RasV12 cells are more than three times faster than HEK-HT cells (Figure 1C). Ras-transformed cells actually migrated in the gel, while normal cells locally jiggled. In this experimental setting, the directionality indicators (d/D, directionality ratio, and direction autocorrelation) [29] were similarly low in both cell types (Figure 1D,E). We concluded that HEK-HT cells and HEK-HT-H-RasV12 cells are good cell models to study how a single oncogenic gene event, the expression of RasV12, stimulates cell migration.

### 3.2. Development of a Micro-Pattern Strategy to Study Spatial-Temporal Localization of Autophagy Organelles during Directional Migration

**Figure 2:**
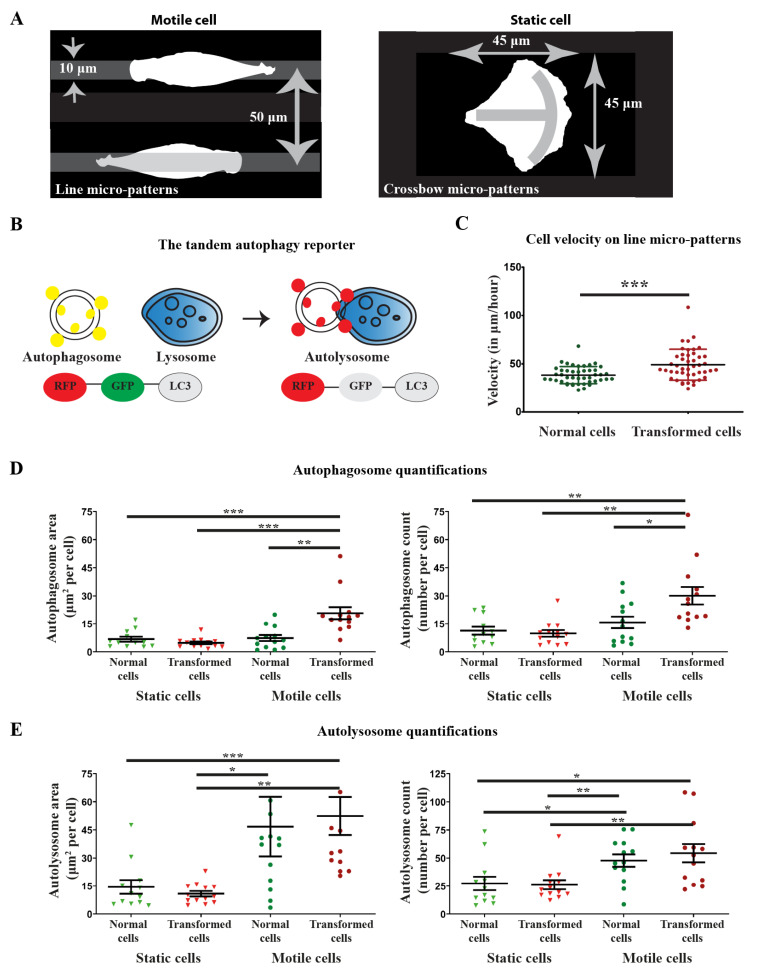
Strategy to study the spatial-temporal localization of LC3 compartments (autophagosomes and autolysosomes) during directional migration. (**A**) Micro-patterns with fibronectin coating were used to force cells to migrate directionally (line micro-pattern) or keep them static (crossbow micro-pattern). (**B**) A tandem reporter RFP-GFP-LC3 was used to monitor the localization of autophagosomes (red + green fluorescence, represented as yellow) and autolysosomes (red fluorescence). After fusion with lysosomes, due to the low pH environment, GFP is quenched and only RFP fluorescence remains. (**C**) Ras-transformed cells (HEK-HT-H-RasV12, in red) are faster on line micro-patterns than isogenic normal cells (HEK-HT, in green). Graph represents mean ± SEM of *n* ≥ 50 cells from 2 independent experiments. (**D**) Quantifications of area (left) and number (right) of autophagosomes per cell, in static and motile cells and in normal and Ras-transformed cells. (**E**) Quantifications of area (left) and number (right) of autolysosomes per cell, in normal and Ras-transformed cells. Graphs represent mean ± SEM of *n* = 12–13 cells per condition from 1 to 4 independent experiments per condition. For each cell, the measurements at 5 different time points (0, 30, 60, 90, and 120 min) were averaged. For statistics, a Student’s *t*-test with unequal variance was used (* *p* value < 0.05, ** *p* value < 0.01, *** *p* value < 0.0001).

Fibronectin micro-patterns were produced on glass coverslips [18], either as 10 μm wide lines to force cells to move directionally or as 45 μm × 45 μm crossbow shapes to keep cells static as control (Figure 2A). Normal HEK-HT cells or transformed HEK-HT-H-RasV12 cells were transiently transfected to express the RFP-GFP-LC3B autophagy reporter [3,22] (Figure 2B), stained with the live nuclear dye SiR-DNA (500 nM) [30,31], seeded on these micro-patterns, and imaged using a spinning disk confocal microscope for 2 h (Movies S3 and S4). The reporter fluorescence was used for cell segmentation, and the nuclear position was used to track cell polarization and migration. Consistently with our results in 3D collagen gels (Figure 1) and with their metastatic potential [23], the Ras-transformed cells migrated faster than isogenic normal cells on the line micro-patterns (Figure 2C).

**Figure 3:**
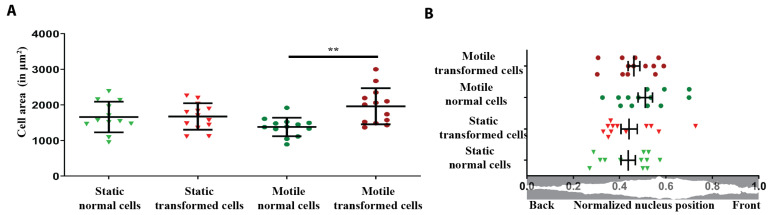
Cell area and position of nuclei in different conditions. (**A**) Measurement of total cell area in different conditions. Each point corresponds to the average area of one cell over 5 time points. (**B**) Location of nucleus with respect to normalized cell length. Each point corresponds to the average of the nucleus positions of one cell over 5 time points. Graphs represent mean ± SEM of 12 cells (in static) and 13 cells (in motile) from 1–4 independent experiments per condition. For each cell, the measurements at 5 different time points (0, 30, 60, 90, and 120 min) were averaged. For statistics, a two-tailed Student’s *t*-test with unequal variance was used (** *p* < 0.01).

No substantial differences between the two cell types were observed as regards the nucleus back–front position with respect to normalized cell length (0.44 ± 0.108, 0.44 ± 0.121, 0.51 ± 0.112, and 0.46 ± 0.0918, for static normal and transformed cells and motile normal and transformed cells, respectively) and the cell average area (1660 ± 430 μm^2^, 1675 ± 372 μm^2^, 1380 ± 261 μm^2^, and 1962 ± 506 μm^2^, for static normal and transformed cells and motile normal and transformed cells, respectively). The only significant difference was between motile transformed cell area and motile normal cell area (see Figure 3).

**Figure 4:**
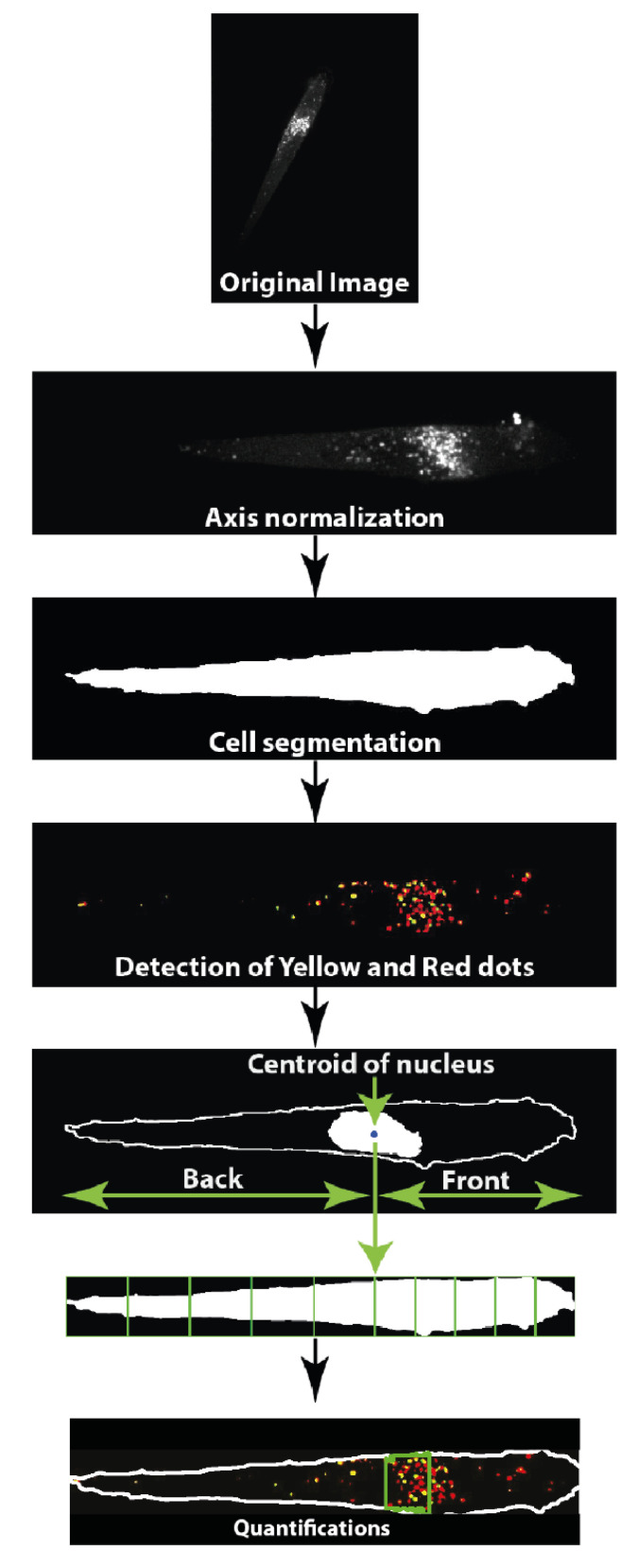
Image analysis workflow of TopoAutophagy computational tool. Images were acquired in four channels: GFP and RFP (for autophagy reporter), far-red (for nuclear staining), and transmission. Axis normalization was performed by aligning the cell along the *x*-axis. Cells were segmented using the whole cell GFP or RFP fluorescence. LC3 compartments were detected in GFP and RFP channels, yellow (red + green) dots corresponding to autophagosomes and red only dots corresponding to autolysosomes. The centroid of the nucleus was measured, and then the cell was partitioned in 10 vertical portions—5 equal portions from nucleus centroid to back cell edge and 5 equal portions from nucleus centroid to front cell edge—for each time point. Yellow and red dots in each cell portion were counted.

An image analysis workflow was automated in an ImageJ environment (TopoAutophagy macro) in order to normalize cell axis; to achieve cell segmentation; to detect the intracellular yellow dots (green and red, i.e., autophagosomes) and red dots (i.e., autolysosomes); to identify the nucleus centroid (at position defined as #5); to partition the cell in 10 vertical ‘portions’ (five back portions with equal width from nucleus centroid to back cell edge [positions #0 to #5] and five front portions with equal width from nucleus centroid to front cell edge [positions #5 to #10]); and to count yellow and red dots in each cell portion (Figure 4). The RFP-GFP-LC3B reporter [3,22] behaved as expected in our HEK-HT cell model: induction of autophagy by nutrient starvation increased the number of red dots, i.e., autolysosomes, while the number of yellow dots, i.e., autophagosomes, remained stable, indicating the expected increase in the autophagy flux (Figure S1).

We compared, in static and motile cells, four autophagy indicators: total area occupied by autophagosomes, number of autophagosomes per cell (Figure 2D), total area occupied by autolysosomes, and number of autolysosomes per cell (Figure 2E). Directed migration globally increased these autophagy indicators, with a more robust migration-dependent stimulation of autophagy in Ras-transformed cells as compared to normal cells.

Area and number of both autophagosomes and autolysosomes were very similar in normal and Ras-transformed cells when immobilized on crossbow micro-patterns, indicating that Ras-transformation does not impact autophagy topology in static cells. Conversely, motile Ras-transformed cells displayed a larger area and a higher number of autophagosomes, but not autolysosomes, as compared to normal cells (Figure 2D,E). We calculated from these measurements the autophagy flux as the ratio of autolysosomes and autophagosomes (Figure S2), i.e., red dots/yellow dots, which is another indicator of autophagy activity. In our experimental setting, the ratio of red to yellow dots appeared similar in normal static and transformed static cells, suggesting that either Ras does not stimulate autophagy flux in cells immobilized on crossbow micro-patters or that the Ras-stimulated rate of autophagosome biogenesis is identical to the rate of reporter degradation in autolysosomes. Moreover, the ratio of red to yellow dots appeared lower in transformed motile cells than in normal motile cells. One possible explanation is that, during directed migration, oncogenic Ras promotes the process of biogenesis of autophagosomes but that their maturation into autolysosomes is faster in normal cells. These hypotheses warrant more biochemical work.

### 3.3. LC3 Compartments Are Polarized at the Front toward the Direction of Migration and Spatially Perturbed by Oncogenic Ras

**Figure 5:**
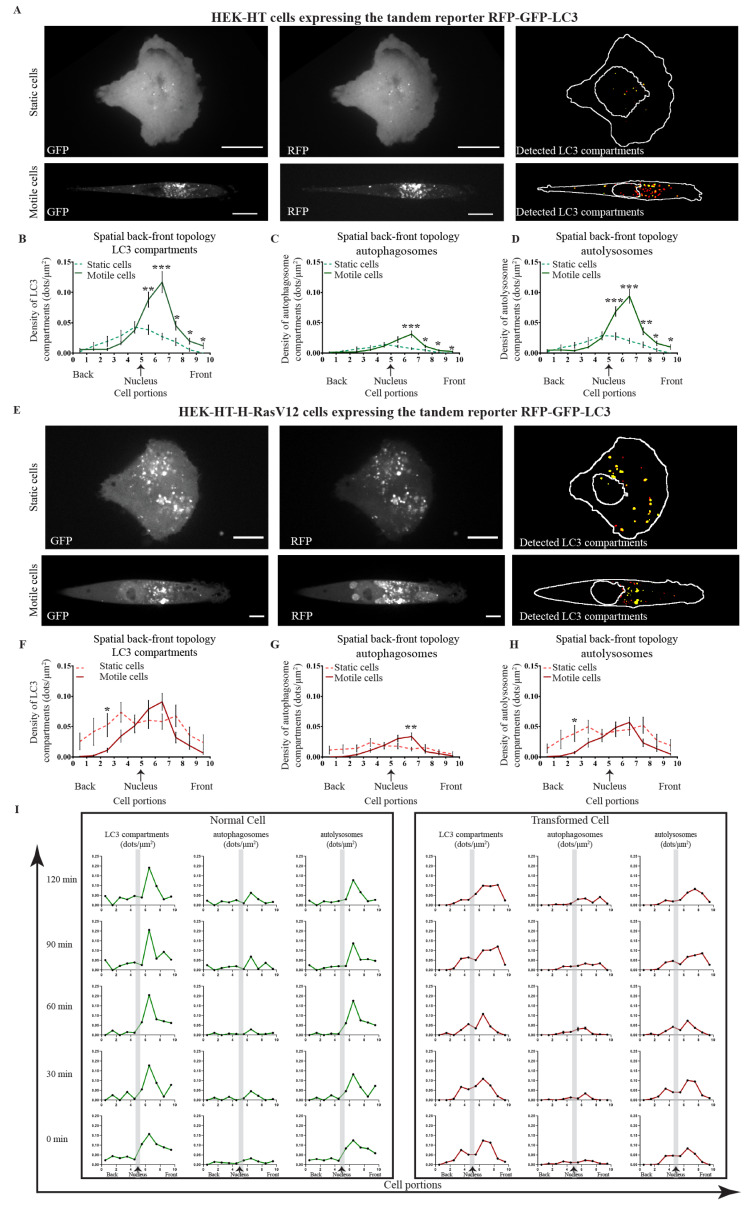
Spatial-temporal localization of LC3 compartments (autophagosomes and autolysosomes) in normal (HEK-HT) and transformed (HEK-HT-H-RasV12), static and motile cells. (**A**) Normal cells. Representative images on crossbow (static) and line (motile) micro-patterns in GFP and RFP channels. The right panel shows the overlay of detected LC3 compartments. (**B**) Normal cells. Spatial back–front topology of LC3 compartment density in static and motile cells. First, the nucleus centroid was identified, and this was further used to define the mid-point of the cell, which was normalized and represented as position #5. (**C**) Normal cells. Spatial back–front topology of autophagosomes in static and motile cells. (**D**) Normal cells. Spatial back–front topology of autolysosomes in static and motile cells. (**E**) Transformed cells. Representative images on crossbow (static) and line (motile) micro-patterns in GFP and RFP channels. The right panel shows the overlay of detected LC3 compartments. (**F**) Transformed cells. Spatial back–front topology of LC3 compartment density in static and motile cells. First, the nucleus centroid was identified, and this was further used to define the mid-point of the cell, which was normalized and represented as position #5. (**G**) Transformed cells. Spatial back–front topology of autophagosomes in static and motile cells. (**H**) Transformed cells. Spatial back–front topology of autolysosomes in static and motile cells. (**I**) Temporal fluctuation of local density of total LC3 compartments, autophagosomes and autolysosomes, in one representative normal cell and one representative transformed cell, during directional motility, at 5 different time points (0, 30, 60, 90, and 120 min). Grey bars correspond to nucleus position. Graphs A–H represent mean ± SEM of *n* = 12–13 cells from 1 to 4 independent experiments per condition. The differences in density values between static and motile cells were statistically tested for each cell portion. For statistics, a two-tailed Student’s *t*-test with unequal variance was used (* *p* < 0.05, ** *p* < 0.01, and *** *p* < 0.001). Scale bars in images are 20 µm.

Localization of LC3 compartments was measured in normal HEK-HT (Figure 5A–D) and transformed HEK-HT-H-RasV12 (Figure 5E–H) during directional migration, with respect to their static controls.

In normal cells, while in the static condition the peak of LC3 compartment density approximately corresponded to the nucleus centroid position (position #5), in the migration condition the majority of LC3 compartments were localized in front of the nucleus (particularly positions #6 and #7), indicating that during migration autophagy is spatially polarized toward the front (Figure 5B). This was observed for both autophagosomes (Figure 5C) and autolysosomes (Figure 5D).

In Ras-transformed cells, the spatial organization of LC3 compartments, both autophagosomes and autolysosomes, was altered (Figure 5F–H). In the static condition, there was no longer a clear central peak of high LC3 compartment density around the nucleus, but a broader distribution along the entire cell length. In the motile condition, LC3 compartments tended to move in front of the nucleus, but they remained more scattered, and the density peaks were much less sharp as compared to normal cells. As a result, while the spatial back–front topology of all LC3 compartments was significantly different between static and motile normal cells, it appeared rather similar in transformed cells.

Next, to characterize the temporal fluctuations of autophagy in directionally migrating cells, the density of all LC3 compartments, and specifically of autophagosomes and autolysosomes, were plotted per cell for each time point (0 min, 30 min, 60 min, 90 min, and 120 min) in normal cells as compared to transformed cells (Figure 5I). In normal cells, the major peak of LC3 compartment density in front of the nucleus was relatively stable over the five time points. On the contrary, in transformed cells the peaks appeared more dynamic, and they moved broadly along the cell length.

How does oncogenic Ras perturb the positioning of autophagy organelles? We speculate that this might occur via some of the many points of connection between the Ras and Rho pathways. In particular, among the three major pathways downstream of Ras, i.e., MAP kinases, PI3 kinases, and Ral GTPases. The latter (specifically via RalB) has been shown to be involved in the regulation of motility/invasion [32] and autophagy [33], suggesting that the RalB GTPase, together with its partners and effectors [32,34], might somehow mechanistically coordinate the interplay between autophagy and motility/invasion in cancer cells, possibly via its cross-talks with Rho GTPases [35,36], particularly in the context of Ras activating mutations.

**Figure 6:**
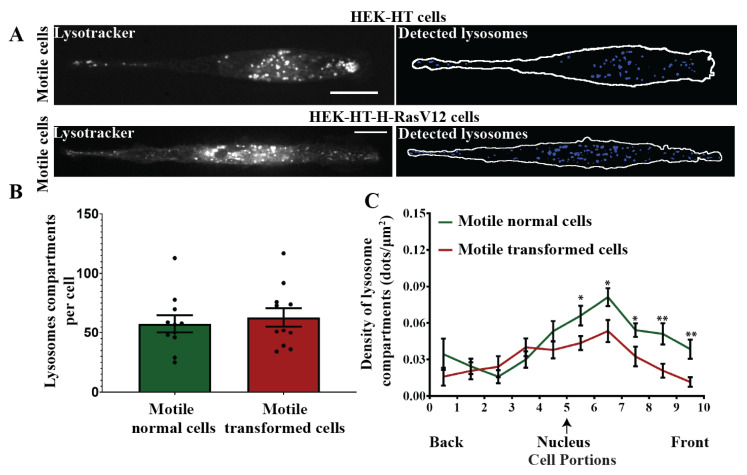
Spatial localization of lysosomes during directional migration: comparison between normal and transformed cells. (**A**) Representative normal HEK-HT and transformed HEK-HT-HRasV12 cells displaying lysotracker staining (left panel) and the segmented lysosomes (right panel). (**B**) Graph represents total lysosome compartments per cell in normal and transformed cells. (**C**) Graph represents spatial back–front topology of lysosome distribution during directional migration in normal and transformed cells. Graphs represent mean ± SEM of *n* = 11 cells per condition from 1 experiment. The differences in lysosome density values between motile normal and motile transformed cells were statistically tested for each cell portion. For statistics, a two-tailed Student’s *t*-test with unequal variance was used (* *p* < 0.05, ** *p* < 0.01). Scale bars in images are 20 µm.

We reasoned that the differential spatial and temporal organization of autophagosomes and autolysosomes between normal and transformed cells might be related to a different spatial organization of lysosomes [37,38]. To explore this hypothesis, we used lysotracker dye to label lysosomes in motile HEK-HT and HEK-HT-H-RasV12 cells seeded on line micro-patterns (Figure 6A). We acquired confocal images in live cells and performed quantifications of number and topology of lysosomes. The number of lysosome compartments was not different between normal and transformed cells (Figure 6B), but, as for autophagosomes and autolysosomes, the front–back distribution of lysosomes was more polarized toward the front in normal cells than in transformed cells (Figure 6C). These results are consistent with the hypothesis that Ras-dependent spatial disorganization of autophagy organelles during motility might be a consequence of altered lysosome positioning, even though the underlying mechanisms remain to be elucidated.

Taken together, our results indicate that when cells start moving, autophagy organelles increase in number and localize in front of the nucleus, suggesting that an active and spatially organized autophagy promotes motility, possibly by locally contributing to focal adhesion turnover [13,14]. However, when RasV12 is expressed, for cells to move faster and to invade, the spatial-temporal organization of autophagy becomes more dispersed, possibly to contribute to the secretion of pro-migratory and pro-invasion factors like metalloproteinases [11,12].

## 4. Conclusions

In conclusion, this work reveals a spatial-temporal organization of the autophagy machinery during directed migration and uncovers an unsuspected perturbation of this organization upon Ras-dependent oncogenic transformation. It is well known that HEK-HT-H-RasV12 cells are more motile, more invasive, and more tumorigenic than HEK-HT cells [23,24,32] (Figure 1, Figure 2C). Here we show that these Ras-transformed cells display a perturbed positioning of autophagosomes, autolysosomes, and lysosomes during cell migration, supporting the notion of an important link between cancer invasion and autophagy topology.

## Data Availability

Data is contained within the article or Supplementary Materials.

## Supplementary Materials

The following are available online at https://www.mdpi.com/article/10.3390/cells10102637/s1, Figure S1: Validation of the tandem RFP-GFP-LC3 autophagy reporter in HEK-HT cells; Figure S2: Calculation of autophagy flux in normal (HEK-HT) and transformed (HEK-HT-H-RasV12), static and motile cells; Movie S1: Normal HEK-HT cell moving in a 3D collagen gel; Movie S2: Transformed HEK-HT-H-RasV12 moving in a 3D collagen gel; Movie S3: Normal HEK-HT cell expressing autophagy reporter and moving on line micro-pattern; Movie S4: Transformed HEK-HT-H-RasV12 cell expressing autophagy reporter and moving on line micro-pattern.
