# Supplementary material for: Autophagy Is Polarized toward Cell Front during Migration and Spatially Perturbed by Oncogenic Ras
Source: Cells. 2021 Oct 2;10(10):2637. doi: 10.3390/cells10102637 (PMC8534269; doi:10.3390/cells10102637)
Supplement: Supplementary file 1 [file cells-10-02637-s001.zip › Supplementary material_rv10-1008.pdf]

Supplementary Figure

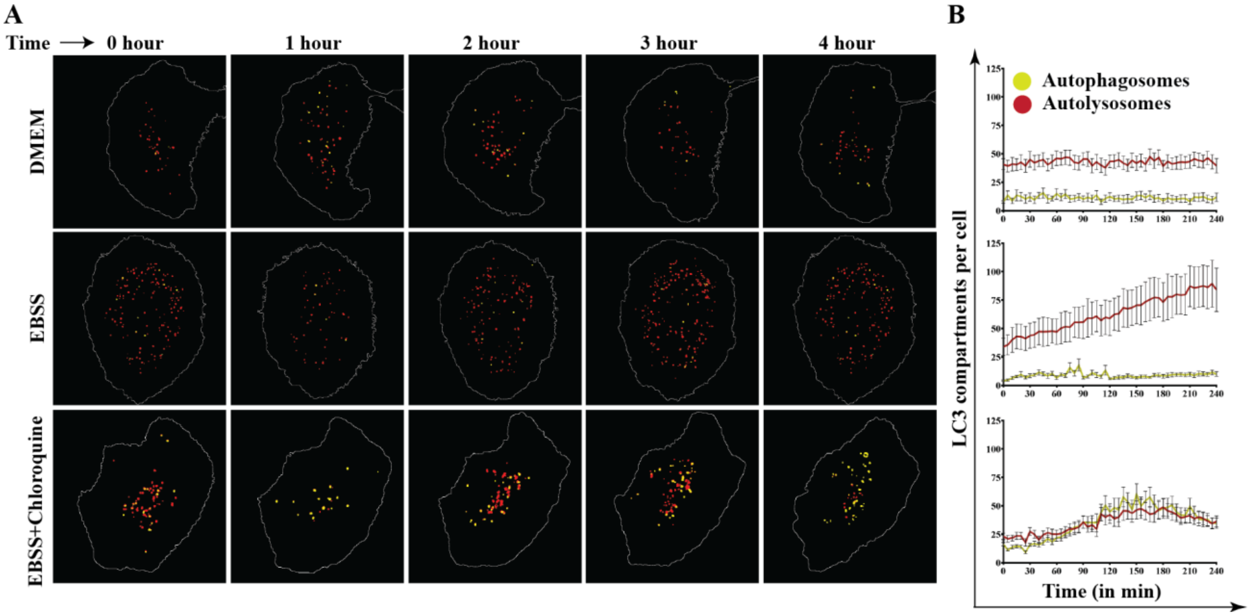

Figure S1. Validation of the tandem RFP-GFP-LC3 autophagy reporter in HEK-HT cells. Cells were grown in rich DMEM medium (top panels), starvation EBSS medium to induce autophagy (middle panels), or in EBSS medium with 50 µM chloroquine to block autophagy flux by inhibiting acidification (bottom panels). (A) Representative images at different time points show detected LC3 compartments, corresponding to autophagosomes (red+green=yellow) and autolysosomes (red only). (B) Quantifications represents mean ± SEM LC3 compartments (autophagosomes in yellow, autolysosomes in red) of 13 cells from 1 experiment. Time 0 corresponds to approximatively 10-15 min after the EBSS medium was changed. Images were acquired every 5 min for 4 hours using spinning disk confocal microscope.

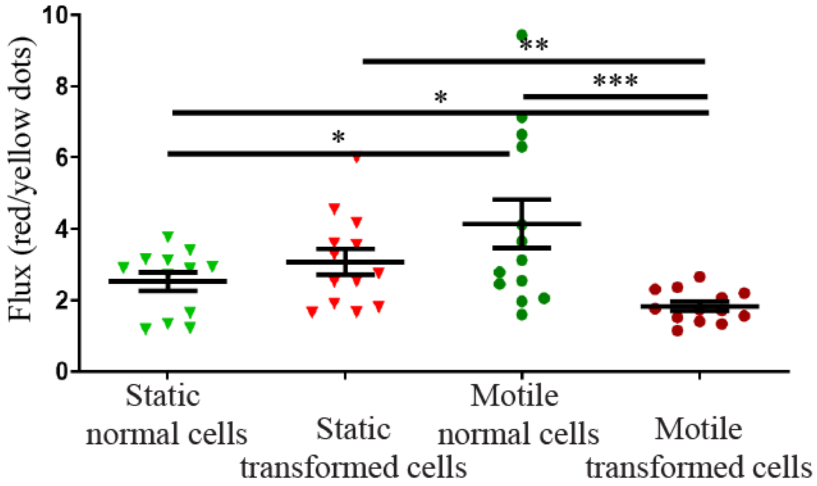

Figure S2. Calculation of autophagy flux in normal (HEK-HT) and transformed (HEK-HT-H-RasV12), static and motile cells.

The autophagy flux is calculated as ratio of autolysosomes and autophagosomes, i.e. red dots/yellow dots, from the measurements reported in Figure 2D and 2E.

Supplementary Movies

Movie S1. Normal HEK-HT cell moving in a 3D collagen gel.
Movie time length is 24 hrs.

Movie S2. Transformed HEK-HT-H-RasV12 moving in a 3D collagen gel.
Movie time length is 24 hrs.

Movie S3. Normal HEK-HT cell expressing autophagy reporter and moving on line micro-pattern.
Cyan color indicates the cell boundary, blue color indicates the cell nucleus, the yellow color dots correspond to the autophagosomes and the red color dots to the autolysosomes. Movie time length is 2 hrs.

Movie S4. Transformed HEK-HT-H-RasV12 cell expressing autophagy reporter and moving on line micro-pattern.
Cyan color indicates the cell boundary, blue color indicates the cell nucleus, the yellow color dots correspond to the autophagosomes and the red color dots to the autolysosomes. Movie time length is 2 hrs.
