# Supplementary figures and images for: Autophagy Is Polarized toward Cell Front during Migration and Spatially Perturbed by Oncogenic Ras
Source: Cells. 2021 Oct 2;10(10):2637. doi: 10.3390/cells10102637 (PMC8534269; doi:10.3390/cells10102637)

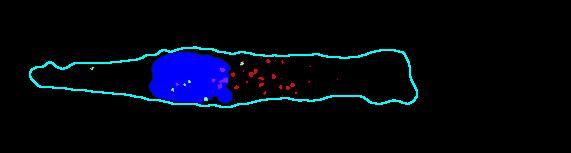

Supplement: Supplementary file 1 [file cells-10-02637-s001.zip › Movie3_Composite_normal.gif]

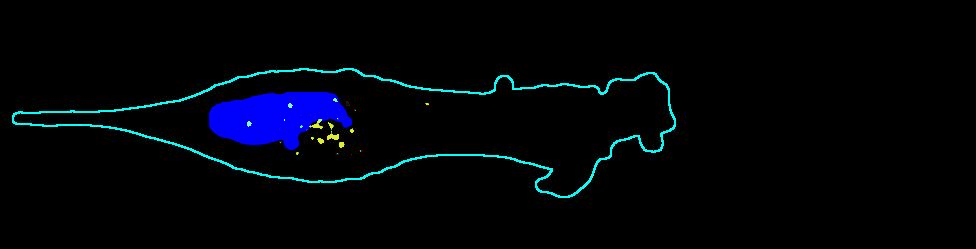

Supplement: Supplementary file 1 [file cells-10-02637-s001.zip › Movie4_Composite_Ras.gif]
